# Management of COVID-19 Outbreak in Argentina: The Beginning

**DOI:** 10.1017/dmp.2020.116

**Published:** 2020-04-22

**Authors:** Nicolás Alejandro Gemelli

**Affiliations:** Adult Intensive Care Unit, Hospital Italiano de Buenos Aires, Argentina

**Keywords:** COVID-19, pandemics, public Health, public Health Surveillance

## Abstract

The aim of this study was to analyze the impact coronavirus disease 2019 (COVID-19) had in Argentina during its initial stage, identify the measures taken to try to mitigate its impact, and briefly compare it with the influenza A H1N1 pandemic in 2009. This is a descriptive study. Pandemics constitute a serious problem to global health with a major impact on the affected countries’ populations. The recent COVID-19 outbreak represents one of the most important viral pandemics lately. It reached Argentina 64 days after the first case was identified in China. Since then, several measures were taken by the Argentinian government to try to mitigate its impact in this initial stage. An updated report of the current situation and its management in different countries is of vital importance regarding global health issues and may serve for feedback and decision-making.

Infectious diseases outbreaks constitute a serious problem to global health with a major impact in countries economy, healthcare systems, and resources. The spread of severe acute respiratory syndrome coronavirus 2 (SARS-CoV-2) has already taken on pandemic proportions, affecting over 100 nations in a matter of weeks. The way in which these outbreaks affect countries depends on multiple factors, and the impact is difficult to foresee. However, updated reports of the current situation are mandatory for local and global scientific communities, the health-care workforce, and government agencies to make decisions based on a dynamic situation analysis.

## REPORT

On December 31, 2019, health authorities in Wuhan City, China, reported a group of pneumonia cases with unknown etiology to the World Health Organization (WHO). On January 9, 2020, the Chinese Center for Disease Control and Prevention identified a new coronavirus as the agent causing this outbreak, currently known as “COVID-19” (coronavirus disease 2019), and on January 30, 2020, the Director-General of the WHO declared the outbreak a public health emergency of international concern, accepting the recommendation of the Emergency Committee of the International Health Regulations.^1^

February 21, 2020, there were already 47 confirmed cases of COVID-19 in Europe with its first fatal case.^2^ Before the illness spread to Argentina, surveillance mechanisms were intensified by the Ministry of Health. Together with this measure, massive media campaigns were launched giving information and establishing guidelines for people to be aware of the disease and its symptoms.

**FIGURE 1:**
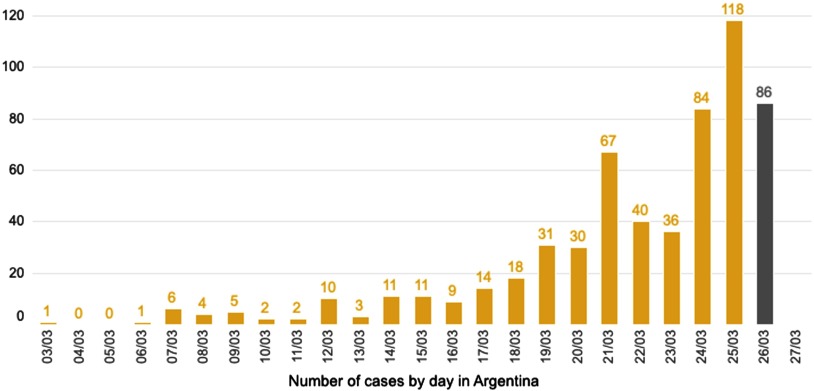
Number of Cases by Day in Argentina.

The first case was confirmed on March 3rd, 64 days after the first case was reported in China. Since then, the number of cases gently ascended (Figure 1); the first death was reported on March 9th. That same day, a special phone line was enabled for general consult and triage. As the number of cases ascended in Argentina and around the world, the epidemiologic controls were progressively intensified. On March 19th, a total of 16 d after the first case was detected in Argentina, an urgent decree was announced by the former president, establishing compulsory national quarantine until March 31st. By means of this law, free circulation of people was forbidden, only allowing certain people to travel to their workplace. Flights were cancelled, and borders were sealed.

Together with the first reports, statistical data were beginning to be collected by official agencies.

On March 25th, 502 cases were reported, with 8 fatal cases. Of the total infected, 41% were female and 59% were male, mainly between 20 and 59 years of age. As a result of the screening, 1946 cases were negative and 1570 were discarded after epidemiologic investigation. Of all confirmed cases, 70 were discharged with follow-up and 2 were discharged with no follow-up.

## DISCUSSION

Even though the infection started to affect people in Wuhan by the end of December 2019, it took just over 2 mo (64 days) to reach Argentina. Compared with the previous pandemic, the 2009 influenza A H1N1 outbreak, it took just 3 wk for H1N1 to get from Mexico to Argentina. A more coordinated global response to the outbreak, together with a greater geographical distance and less frequency of flights from China to Argentina than from Mexico to Argentina might have had a major impact on the speed at which the virus spread. This factor definitely gave Argentinians the opportunity to design containment plans that could mitigate the spread.

Considering most of the cases were imported, canceling flights, instituting quarantine, and closing the borders were the most important decisions taken; however, they will probably have a severe economic impact. These measures were not taken during the H1N1 outbreak and could have affected the rate of transmission for that outbreak.

In relation to the actual number of affected people in the course of this pandemic, the lack of validated diagnostic tests, the delay in the samples’ processing, and the absence of proper health-care facilities in smaller cities may have resulted in an under-estimate of the total number of positive cases.

**FIGURE 2:**
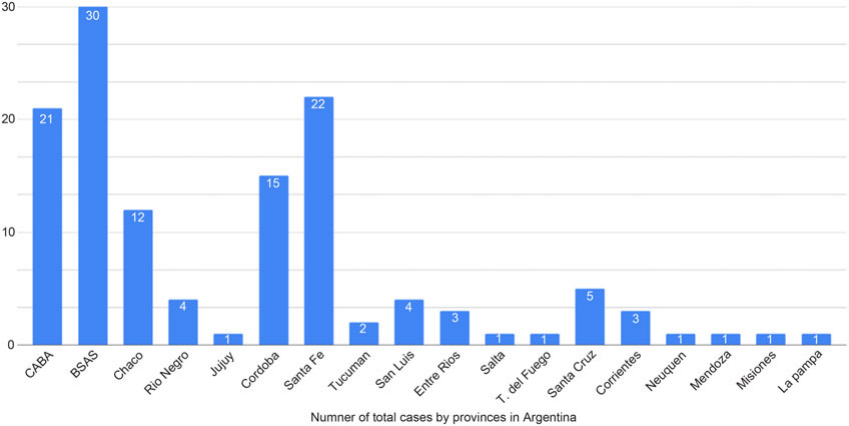
Number of Total Cases by Provinces in Argentina.

Similar to what we are experiencing currently (Figure 2), the spatial distribution of cases of the influenza A H1N1 pandemic followed a hierarchical pattern along the main cities of Buenos Aires, Cordoba, and Santa Fe province. The main entry point of individuals from affected countries to South America is through Ezeiza International Airport located in Capital City (Ciudad Autonoma de Buenos Aires).^3^ From May to December 4, 2009, a total of 11,234 H1N1 infections were confirmed in Argentina, and 613 (5.5%) infected patients died. In the Americas, Argentina had the fourth highest number of deaths associated with influenza virus subtype H1N1, after the United States, Brazil, and Mexico.^4,5,6^

We still need time to know the real impact COVID-19 will have on our country. For this reason, it is very important we keep collecting and sharing data for further analysis.

## CONCLUSION

An updated report of the outbreak situation and management in different countries with diverse backgrounds is of vital importance regarding global health issues and may also serve for feedback and decision-making.

We still have to figure out what this COVID-19 outbreak will teach us and what future strategies could be implemented to help mitigate the great impact pandemics have on developing countries.
